# A clinical trial to validate event-related potential markers of Alzheimer's disease in outpatient settings

**DOI:** 10.1016/j.dadm.2015.08.004

**Published:** 2015-10-02

**Authors:** Marco Cecchi, Dennis K. Moore, Carl H. Sadowsky, Paul R. Solomon, P. Murali Doraiswamy, Charles D. Smith, Gregory A. Jicha, Andrew E. Budson, Steven E. Arnold, Kalford C. Fadem

**Affiliations:** a Neuronetrix, Louisville, KY, USA; b Department of Neurology, Nova Southeastern University, Fort Lauderdale, FL, USA; c Department of Psychology, Williams College, Williamstown, MA, USA; d Departments of Psychiatry and Medicine, Duke Medicine and Duke Institute for Brain Sciences, Durham, NC, USA; e Department of Neurology, University of Kentucky, Lexington, KY, USA; f Department of Cognitive & Behavioral Neurology, VA Boston Healthcare System, Boston, MA, USA; g Departments of Psychiatry and Neurology, University of Pennsylvania, Philadelphia, PA, USA

**Keywords:** Multicenter clinical trial, Event-related potentials, Oddball paradigm, Early stage Alzheimer's disease, Outpatient settings, Automated ERP data analysis

## Abstract

**Introduction:**

We investigated whether event-related potentials (ERP) collected in outpatient settings and analyzed with standardized methods can provide a sensitive and reliable measure of the cognitive deficits associated with early Alzheimer's disease (AD).

**Methods:**

A total of 103 subjects with probable mild AD and 101 healthy controls were recruited at seven clinical study sites. Subjects were tested using an auditory oddball ERP paradigm.

**Results:**

Subjects with mild AD showed lower amplitude and increased latency for ERP features associated with attention, working memory, and executive function. These subjects also had decreased accuracy and longer reaction time in the target detection task associated with the ERP test.

**Discussion:**

Analysis of ERP data showed significant changes in subjects with mild AD that are consistent with the cognitive deficits found in this population. The use of an integrated hardware/software system for data acquisition and automated data analysis methods make administration of ERP tests practical in outpatient settings.

## 1 Background

Despite the emergence of putative biomarkers for Alzheimer's disease (AD) [1], clinical diagnostic accuracy is suboptimal [2]. A sensitive and reliable physiological measure of the cognitive deficits associated with AD could provide insight in the cognitive physiology of the disease, and help with diagnosis, and assessment of severity and progression.

Event-related potentials (ERP) reflect well-characterized brain responses to sensory, motor, and cognitive events [3]. As such, ERP methods are well suited to detect and quantify the cognitive deficits associated with AD [4]. ERP have been found to be altered in AD beginning in the very early stages of the disease. ERP tests on young presymptomatic individuals who carry mutations in the presenilin-1, and amyloid precursor protein genes show significant changes in ERP patterns years before the onset of behavioral symptoms and the development of AD [5], [6]. ERP have shown potential utility as biomarkers of disease progression and subsequent conversion to dementia in individuals with mild cognitive impairment (MCI). ERP responses to auditory stimuli contain discriminative information that predicts which MCI patients are likely to progress to AD [7], and patients with amnestic MCI that are at high risk of conversion to AD have abnormal ERP during a word repetition task [8]. ERP have also been shown to reliably track the cognitive decline associated with AD progression. ERP markers of cognitive function are increasingly altered in longitudinal studies on MCI and AD patients [9], [10]. Finally, ERP are sensitive to the effects of cognitive enhancers currently used for the treatment of AD. ERP measures are reliable instruments for the assessment of the cognitive response to cholinesterase inhibitors such as donepezil, while the effects of the selective N-methyl-D-aspartate (NMDA) antagonist memantine on ERP correlate with changes in mini-mental state examination (MMSE) score [11], [12], [13].

Although the potential of ERP as a sensitive and reliable cognitive biomarker for AD has been known for a long time (for review, see [14], [15], [16]), the promise of this technique has not been yet fully realized through wide adoption of ERP in clinical use. Primary reasons have been the lack of standardization of ERP acquisition and data analysis techniques, and the impracticality of conducting ERP tests in clinical environments on actual patients. Recent advances in electronics and analysis algorithms have made it possible to administer ERP tests in a practical manner. There is now a need for large population-based studies that can confirm the usefulness of ERP as cognitive biomarkers for AD outside the laboratory [6].

In our multicenter clinical study, we investigated whether ERP collected in an outpatient setting and analyzed with automated, standardized methods can achieve results equivalent to those reported from academic laboratories and provide a sensitive and reliable measure of the cognitive deficits associated with early AD.

## 2 Materials and methods

### 2.1 Study participants

A total of 103 subjects with probable mild AD and 101 healthy controls (HC) aged between 60 and 90 years were recruited at seven clinical study sites. The study (ClinicalTrials.gov number NCT00938665) was approved by institutional review boards for each site, and a written informed consent was obtained from each study participant.

### 2.2 Subjects screening

All study subjects received a thorough medical history and neurologic examination. General inclusion criteria for the study included a modified Hachinski score ≤4 and a geriatric depression scale (GDS) short form score ≤5. Exclusion criteria were the use of antidepressants other than selective serotonin uptake inhibitors, major psychiatric disorders, and clinically significant neurologic diseases other than AD. Subjects taking sedatives and/or memory dietary supplements were asked to suspend them for the 72 hours before screening and testing.

The diagnosis of probable AD was made on the basis of the National Institute of Neurological and Communication Disorders and the Stroke-Alzheimer's Disease and Related Disorders Association criteria [17]. The inclusion criteria for the AD cohort were designed to recruit subjects in the early stages of the disease and encompassed an MMSE score between 21 and 26, a clinical dementia rating (CDR) score of 0.5, 1, or 2, and an education adjusted score on the delayed recall of the Wechsler logical memory II subscale of ≤3 for 0–7 years of education, ≤5 for 8–15 years of education, and ≤9 for 16 or more years of education.

Inclusion criteria for the HC cohort were an MMSE score of 27 and above, a CDR score of 0, and an education adjusted score on the delayed recall of the Wechsler logical memory II subscale of ≥4 for 0–7 years of education, ≥6 for 8–15 years of education, and ≥10 for 16 or more years of education.

### 2.3 Experimental paradigm

Subjects who met inclusion criteria at screening were tested using a three-stimulus oddball paradigm (for review, see [18], [19]).

Stimuli comprised of standard tones (1000 Hz), target tones (2000 Hz), and unexpected distractor tones (white noise) that were played with probabilities of .75, .15, and .10. Tones were presented in pseudorandom order, so that target and distractor tones were never presented sequentially [20]. Subjects were instructed to respond to the target stimuli by pressing a button with their dominant hand. For each test, between 300 and 400 stimuli were presented binaurally through insert ear phones at 70-dB volume. The tone duration for each stimulus was 100 ms with rise and fall times of 10 ms. The interstimulus interval was randomized between 1.5 and 2 s. During the test, subjects sat comfortably in a chair in an office room under regular lighting conditions. One HC and four mild AD subjects who were unable to follow instructions were excluded from all statistical analyses.

### 2.4 Testing procedures and data analysis

Electroencephalographic (EEG) activity was recorded from 7 electrode sites (Fz, Cz, Pz, F3, P3, F4, and P4) of the international 10-20 system [21] using a COGNISION Headset (Neuronetrix). Electrodes were referenced to averaged mastoids (M1, M2), and Fpz served as the common electrode. The headset used for data collection has been validated to perform reliable ERP recordings when skin contact impedance is <70 kΩ, a practical requirement for recording in standard office environments. Impedance was automatically checked at all electrodes after each target or distractor tone, and was kept below this limit throughout each test. Data were collected from −240 to 1000 ms around the stimuli, digitized at 125 Hz, and bandpass filtered from 0.3 to 35 Hz. An automatic artifact threshold detection limit of ±100 μV was set for the tests. Trial sets of a deviant tone and the immediately preceding standard tones (epoch sets) with artifacts exceeding the threshold were rejected in real time and immediately repeated.

Trial averaging and extraction of ERP measures were automatically performed by the COGNISION System software (Neuronetrix). EEG data from each trial were baseline corrected using the prestimulus period [6], [22] and averaged according to stimulus. For standard tones, only the trials immediately preceding target and distractor stimuli were averaged. During data preprocessing, recordings that exceeded two times the root mean square value (RMS) for the EEG test data or with wrong button presses were rejected and excluded from averaging. ERP waves that averaged less than 20 trials after preprocessing were eliminated from all analyses [23].

**Fig. 1:**
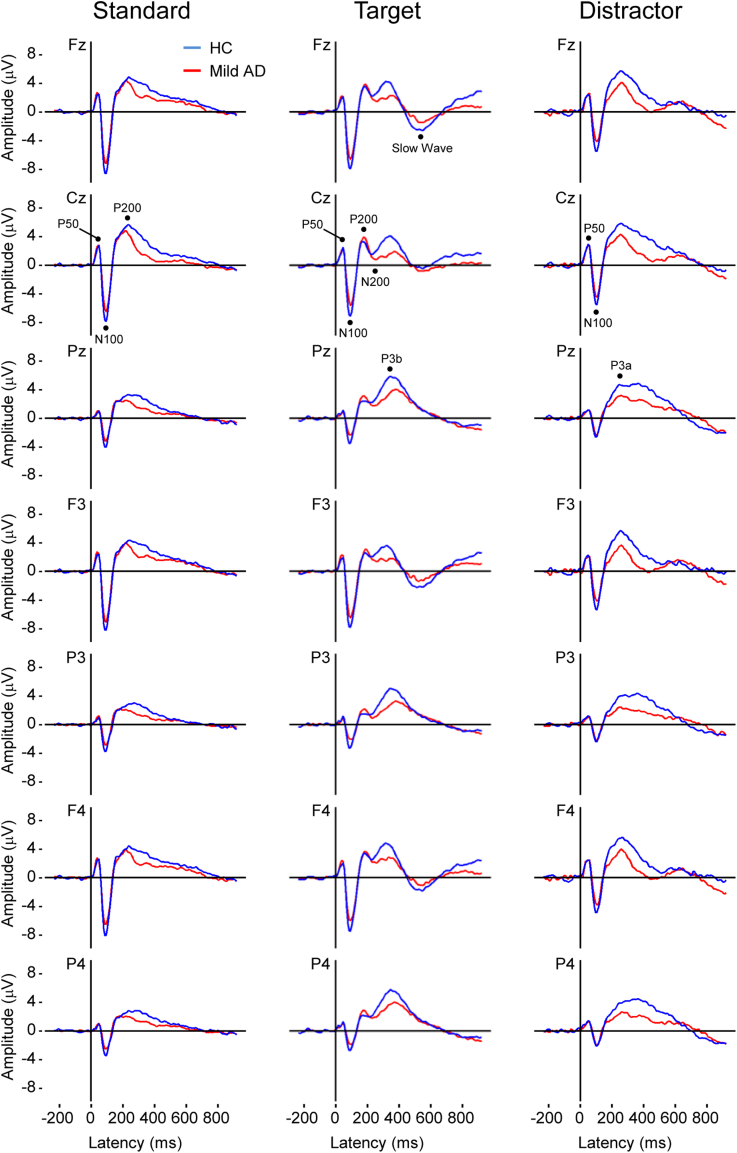
Grand average ERPs for standard, target, and distractor stimuli in mild AD and HC subjects. ERP features for each stimulus are shown at the electrode site where they were found to be more prominent. Abbreviations: ERP, event-related potentials; HC, healthy controls; AD, Alzheimer's disease.

Peak amplitude of the ERP features was measured as the difference between the mean prestimulus baseline and maximum peak amplitude. Peak latency was defined as the time point corresponding to the maximum amplitude and was calculated relative to stimulus onset [24], [25]. P50 and N100 were measured from all stimuli. P200 was measured from standard and target tones. N200, P3b, and slow wave were measured from the target tone and P3a from the distractor tone (Fig. 1).

The P50 ERP feature was defined as the maximum positivity between 24 and 72 ms poststimulus, N100 was the maximum negativity between 70 and 130 ms, P200 the maximum positivity between 180 and 235 ms, and N200 the maximum negativity between 205 and 315 ms. The P3a was defined as the maximum positivity between 325 and 500 ms, and the P3b as the maximum positivity between 325 and 580 ms. Finally, the slow wave was the maximum negativity between 460 and 680 ms. All time windows were determined by inspecting individual averages and group grand averages [26].

The feature extraction algorithm used for the analysis defined a maximum positivity as the highest point in the measurement window that was surrounded on both sides by lower voltage. If a maximum positivity was not present in the time window chosen for an ERP feature, the algorithm would not select a peak for that channel.

Together with peaks amplitude and latency, the algorithm for data analysis also calculated mean amplitude for the ERP features of interest, defined as the average voltage over the specified measurement window for each ERP feature [27].

Finally, accuracy and reaction time of button presses were also analyzed. Accuracy was calculated as the percent of correct responses to target tones, whereas false alarms indicated button presses to nontargets. Reaction time was calculated as the time from stimulus onset to button press. Median reaction times were calculated for each subject to limit the influence of any outlier reaction times [5].

### 2.5 Statistical analysis

Group comparisons were analyzed using χ^2^ test and Student *t* test for categorical and quantitative variables, respectively. Age was significantly different between mild AD and HC groups and was used as a covariate in all statistical comparisons where data correlated with age. *P* values <.05 were considered significant. When multiple comparisons were performed, a Bonferroni correction was applied to control for type I error and the adjusted *P* values were reported.

Correlations between ERP component values were analyzed using Pearson correlation coefficients.

## 3 Results

### 3.1 Demographics and clinical data

There were no significant differences in gender and education between study groups. Age, however, was higher on average in subjects with mild AD (*t* = 2.94, *P* < .05).

Statistical comparison of clinical data between groups showed, as expected, lower MMSE (*t* = −28.93, *P* < .01), lower Wechsler logical memory (*t* = −20.28, *P* < .01 and *t* = −28.38, *P* < .01 for immediate and delayed recall, respectively), and higher CDR (*t* = 30.54, *P* < .01) scores in subjects with mild AD. These subjects also had a higher GDS (*t* = 7.32, *P* < .01), whereas the Hachinski score was similar between groups (Table 1).

### 3.2 ERP test

Morphology of the grand average waves for standard, target, and distractor stimuli was different between groups (Fig. 1). The differences were larger for target and distractor tones, and for the late cognitive responses than for the early sensory measures (Supplementary Fig. 1).

Statistical comparisons for ERP features in mild AD versus HC are listed in Table 2. Age correlated with N100, P3b, and P3a latency measures (R^2^ >0.05), and was used as covariate in comparisons for these ERP measures.

Analysis of ERP features for the standard tone showed lower N100 amplitude (*t* = 6.25, *P* < .01) and P200 amplitude (*t* = −3.39, *P* < .01) in the mild AD group. This group of subjects also had higher P50 (*t* = 3.68, *P* < .01) and lower N100 average amplitudes (*t* = 5.50, *P* < .01) than HC.

Comparisons for the target tone indicated that subjects with mild AD had lower N100 amplitude (*t* = 4.88, *P* < .01), lower P3b amplitude (*t* = −5.65, *P* < .01), and a more negative N200 peak (*t* = −3.38, *P* < .01) than HC. This group of subjects also showed a delay in the late cognitive measures with longer latencies for the N200 (*t* = 3.43, *P* < .01), P3b (*t* = 2.66, *P* < .05), and slow wave (*t* = 2.88, *P* < .05). Finally, data for average amplitude showed lower N100 (*t* = 4.99, *P* < .01), lower N200 (*t* = −4.73, *P* < .01), and a tendency to lower P3b (*t* = −2.47, *P* < .1) in subjects with mild AD, thus closely resembling amplitude data.

When ERP features for the distractor tone were analyzed, statistical comparisons showed decreased amplitude (*t* = −2.55, *P* < .05) and longer latency (*t* = 2.83, *P* < .05) for the P50 in subjects with mild AD. These subjects also had smaller N100 and P3a amplitudes (*t* = 4.24, *P* < .01 and *t* = −8.07, *P* < .01) and average amplitudes (*t* = 3.57, *P* < .01 and *t* = −10.68, *P* < .01, respectively).

A follow-up single-channel analysis at midline electrodes for the ERP features that were statistically different between groups showed that changes in N100 measures were pronounced at the frontal and central electrode sites, whereas changes in P3a and P3b ERP features were more prominent at the central and parietal electrodes (Table 3).

When performance in the target detection task associated with the ERP test was analyzed, there were significant differences between groups. Subjects with mild AD had a lower percentage of correct responses to target tones (*t* = −4.61, *P* < .01), a higher number of button presses to nontargets (*t* = 3.43, *P* < .01), and a longer reaction time for accurate button presses than HC (*t* = 2.4, *P* < .05; Table 4).

Finally, analysis of correlations across ERP features that were significantly different between groups showed overall low Pearson coefficients. Exceptions were N100 measures across different stimuli, and correlations among amplitude and average amplitude for the same ERP measure (Supplementary Table 1).

## 4 Discussion

Study results showed significant differences in ERP measures between subjects with mild AD and HC. The group differences included both ERP features extracted from the average waves for the test stimuli and behavioral measures from the target detection task.

The most widely investigated and best understood changes in ERP in mild AD are related to the P3b, or classic P300. This ERP feature is elicited when a deviant stimulus is associated with a task and reflects an update in working memory (for review of the neuropsychological origins of the P3b, please see [28]). The P3b amplitude is determined by the amount of attentional resources allocated when working memory is updated [29]. The P3b latency reflects stimulus evaluation and classification speed [30], [31]. The majority of studies that have looked at differences in P3b between AD subjects and HC have found that P3b amplitude was typically smaller, and P3b latency was longer in subjects with AD (for an overview, please see [32]). Consistent with our results, when subjects were administered an auditory oddball paradigm where discrimination of standard and target tones was easy, group differences were larger for P3b amplitude than latency [32].

Together with the P3b, other significant changes in the ERP wave for the target tone included longer latencies for the N200 and slow wave, and a more negative N200 in subjects with mild AD.

The N200 is a negative peak that immediately precedes the P3b. This ERP feature is linked to the cognitive processes of stimulus identification and distinction [33] and its peak latency has been shown to correlate with measures of executive function and attention [34]. Published studies have reported delayed latency [34] and smaller amplitude [9] for the N200 in AD. Indeed, N200 latency has proven useful in separating AD subjects from subjects with MCI and HC [34], and N200 amplitude has been used in combination with P300 latency to track longitudinal changes in overall cognitive function in MCI [9]. Our findings offer further evidence that both the peak latency and amplitude are affected in AD.

The slow wave is a negative deflection that follows the P3b. This ERP feature has frontal and central scalp distribution [5] and reflects a final stage of stimulus evaluation [35]. The slow wave amplitude correlates with task demands and it is inversely correlated to stimulus detection accuracy, suggesting that an increase in peak amplitude might reflect the need for further stimulus processing. The slow wave latency is affected by task difficulty, and the relative ease of categorizing events in an oddball test probably accounts for the early onset and short duration of the slow wave in this ERP paradigm [35]. In our study, slow wave latency was delayed in mild AD. Our data are consistent with a previous report of increased slow wave latency in MCI [9] and suggest that AD subjects might require more time for stimulus processing than HC.

Contrary to the P3b, reports on the effects of AD and other dementias on P3a amplitude and latency are scarce, and the findings have been to some extent inconsistent [26], [36], [37]. The P3a is associated with engagement of attention and processing of novelty [28]. The peak amplitude is a measure of focal attention and has been shown to positively correlate with executive function [38]. The P3a latency reflects orientation to a nontarget deviant stimulus [16]. Our data show a reduction of P3a amplitude in subjects with mild AD that is consistent with reports of decreased attention and executive function in neuropsychological testing in this population [39]. Moreover, the large group differences in P3a amplitude together with reports of a decline in attention and some executive skills very early in the disease [40], [41] suggest that this ERP feature could be a useful measure of cognitive deficit from the preclinical stage of AD.

Like for the P3a, N100 amplitude also showed a large decrease in the mild AD group. The effect was present in response to all stimuli. Although the N100 reflects bottom-up information such as stimulus characteristics [42], [43], it is modulated by attention and memory-related variables [44], [45]. Thus, it is possible that the lower amplitude of the N100 in subjects with mild AD might reflect attention and memory deficits in these subjects. Indeed, neuropathologic studies show that sensory cortices are typically spared until the advanced stages of AD [46]. A decrease in N100 amplitude could reflect changes in regulatory inputs from brain regions that are involved in higher cognitive processes and are more directly affected by the disease in its early stages. For example, the prefrontal cortex and the nucleus basalis have been shown to modulate auditory cortical responses to sound [47], [48].

In addition to changes in the ERP wave, subjects with mild AD also showed decreased performance in the behavioral task associated with the ERP test. This group of subjects had lower button press accuracy and longer reaction time. Previous work by Polich and Corey-Bloom [32] has shown increased response time and error rate in AD patients across different auditory and visual oddball paradigms. Our data confirm these findings in patients tested in outpatient settings and suggest that results from the behavioral task of the ERP test could help discriminate subjects with mild AD from healthy aging.

Scientific literature on the neuropsychology of ERP measures indicates that the different ERP features collected with an oddball paradigm provide complimentary information [49]. Indeed, in our study, correlations between ERP features that showed significant group differences were overall quite low, suggesting that data from a single test can be used to assess deficits in several cognitive domains affected by AD. Automated data analysis methods such as the ones used in the present study make extraction of multiple ERP features from data sets practical, thus providing a fast and reliable method to look at multiple sensory and cognitive measures.

Although correlations across different ERP features were generally low, correlations between each feature amplitude and average amplitude were high. These data suggest that an ERP feature average amplitude likely shares the same functional interpretation with its amplitude, and can be used as a proxy measure to confirm amplitude data, or in situations where an ERP peak might be difficult to identify.

## 5 Conclusion

Analysis of data collected from this large multicenter study closely reflects findings reported from research laboratories on changes in ERP measures in subjects with AD. In addition, the study also offers insights on additional ERP differences in HC versus mild AD that to our knowledge had not yet been reported, or for which there did not seem to be a consensus. Follow-ups will include a classification-based analysis to measure sensitivity and specificity of ERP in diagnosing subjects with early AD in outpatient settings and a separate study to correlate ERP measures with neuropsychological tests that are widely used to assess cognitive status.

Data for the study were collected in outpatient settings from nonspecialized personnel. Our results suggest that the use of an integrated hardware/software system for ERP testing and automated data analysis tools can address the practical limitations that have hindered a wide adoption of electrophysiological measures as useful biomarkers for AD outside research laboratories.

**Research in context**

1. Systematic review: Since the 1970s, a large scientific literature has shown that event-related potentials (ERP) can provide a sensitive, physiological measure of the cognitive deficits associated with Alzheimer's disease (AD). However, the use of ERP in AD has been mostly limited to studies conducted at a single site and/or on a limited number of subjects. There is a need for large population-based studies that can confirm the usefulness of ERP as biomarkers for AD in outpatient settings.
2. Interpretation: Findings from our multicenter clinical study show that ERP collected with standard methods and analyzed using automated data analysis tools provide a sensitive and practical measure of the cognitive deficits associated with early AD.
3. Future directions: Additional analysis of the ERP data from the study, using classification-based machine learning approaches, will provide further insight on the sensitivity and specificity of ERP in diagnosing subjects with early AD in outpatient settings.

## Figures and Tables

**Table 1:** Demographic and clinical characteristics of study participants

Characteristics	HC (n = 100)	AD (n = 99)
Age	73.2 ± 0.71	76.2 ± 0.74^∗^
Male (%)	40	48.5
Education (y)	14.9 ± 0.29	14.4 ± 0.32
MMSE	29.1 ± 0.08	23.4 ± 0.19^∗∗^
CDR	0.0 ± 0.0	0.9 ± 0.03^∗∗^
WMS-R logical memory		
Immediate recall	14.6 ± 0.31	5.3 ± 0.33^∗∗^
Delayed recall	13.7 ± 0.33	2.1 ± 0.24^∗∗^
GDS	0.8 ± 0.11	2.3 ± 0.16^∗∗^
Hachinski	0.6 ± 0.07	0.6 ± 0.07

Abbreviations: HC, healthy controls; AD, mild Alzheimer's disease; MMSE, mini-mental state examination; CDR, clinical dementia rating; WMS-R, Wechsler memory scale-revised; GDS, geriatric depression scale; SEM, standard error of the mean.

NOTE. Data are represented as mean ± SEM. *P < .05 and **P < .01 compared with HC after Bonferroni correction.

**Table 2:** ERP features in HC and mild AD

ERP feature	Stimulus	Amplitude (μV)		Latency (ms)		Average amplitude (μV)	
HC	AD	HC	AD	HC	AD
P50	Standard	2.77 ± 0.08	2.95 ± 0.08	44.8 ± 0.4	44.3 ± 0.4	0.29 ± 0.06	0.60 ± 0.06^∗∗^
N100	Standard	−7.23 ± 0.14	−6.00 ± 0.14^∗∗^	93.0 ± 0.4	95.2 ± 0.5	−4.56 ± 0.11	−3.73 ± 0.11^∗∗^
P200	Standard	5.26 ± 0.14	4.64 ± 0.12^∗∗^	214.5 ± 1.0	211.7 ± 0.8	3.44 ± 0.11	3.14 ± 0.10
P50	Target	2.79 ± 0.09	2.79 ± 0.09	42.6 ± 0.5	43.2 ± 0.5	0.36 ± 0.06	0.52 ± 0.07
N100	Target	−6.64 ± 0.14	−5.63 ± 0.15^∗∗^	95.2 ± 0.5	98.6 ± 0.6	−4.25 ± 0.12	−3.43 ± 0.12^∗∗^
P200	Target	4.49 ± 0.18	4.86 ± 0.18	202.8 ± 0.9	201.2 ± 0.9	2.35 ± 0.13	2.61 ± 0.14
N200	Target	−0.31 ± 0.17	−1.10 ± 0.16^∗∗^	251.1 ± 1.3	257.9 ± 1.5^∗∗^	2.84 ± 0.14	1.93 ± 0.13^∗∗^
P3b	Target	6.03 ± 0.20	4.42 ± 0.20^∗∗^	396.0 ± 2.8	419.6 ± 3.3^∗^	1.92 ± 0.16	1.40 ± 0.13∼
Slow wave	Target	−2.54 ± 0.20	−2.65 ± 0.18	563.6 ± 2.5	575.4 ± 3.2^∗^	−0.02 ± 0.15	0.19 ± 0.15
P50	Distractor	3.70 ± 0.09	3.35 ± 0.10^∗^	45.2 ± 0.6	47.6 ± 0.6^∗^	1.18 ± 0.08	1.26 ± 0.09
N100	Distractor	−5.34 ± 0.14	−4.47 ± 0.15^∗∗^	101.1 ± 0.5	103.9 ± 0.5	−2.84 ± 0.11	−2.21 ± 0.12^∗∗^
P3a	Distractor	5.88 ± 0.19	3.63 ± 0.20^∗∗^	417.3 ± 2.4	419.8 ± 3.0	3.40 ± 0.15	1.26 ± 0.13^∗∗^

Abbreviations: ERP, event-related potentials; HC, healthy controls; AD, mild Alzheimer's disease; SEM, standard error of the mean.

NOTE. Data are represented as mean ± SEM. ∼*P* < .1; *P < .05; and **P < .01 compared to HC after Bonferroni correction.

**Table 3:** Statistically significant differences between groups at single midline electrodes

ERP feature	Stimulus	Type	Loc.	HC	AD	*P* value
P3a	Distractor	Av. Ampl.	Cz	4.03 ± 0.42	1.32 ± 0.35	<.001
P3a	Distractor	Av. Ampl.	Pz	4.31 ± 0.34	2.22 ± 0.32	<.001
P3a	Distractor	Amplitude	Cz	6.96 ± 0.50	3.98 ± 0.49	<.001
P3a	Distractor	Amplitude	Pz	7.01 ± 0.42	4.54 ± 0.46	<.001
N100	Standard	Amplitude	Fz	−9.69 ± 0.31	−8.01 ± 0.35	.001
N100	Standard	Amplitude	Cz	−8.78 ± 0.33	−7.20 ± 0.32	.002
P3a	Distractor	Av. Ampl.	Fz	2.76 ± 0.42	0.77 ± 0.41	.003
N100	Standard	Av. Ampl.	Fz	−6.47 ± 0.24	−5.35 ± 0.28	.007
P3b	Target	Amplitude	Pz	7.36 ± 0.39	5.74 ± 0.38	.010
N100	Standard	Av. Ampl.	Cz	−5.64 ± 0.24	−4.60 ± 0.26	.012
N100	Target	Av. Ampl.	Cz	−5.26 ± 0.28	−4.12 ± 0.29	.015
N100	Standard	Amplitude	Pz	−4.82 ± 0.26	−3.87 ± 0.24	.025
N100	Target	Amplitude	Cz	−8.03 ± 0.37	−6.74 ± 0.36	.028
N100	Target	Amplitude	Pz	−4.32 ± 0.25	−3.39 ± 0.26	.034
N100	Distractor	Amplitude	Fz	−6.85 ± 0.39	−5.39 ± 0.44	.039
P3b	Target	Latency	Pz	395.8 ± 6.2	419.3 ± 7.6	.049

Abbreviations: Loc., electrode location according to the 10/20 system; HC, healthy controls; AD, mild Alzheimer's disease; Av. Ampl, average amplitude; SEM, standard error of the mean.

NOTE. Data are represented as mean ± SEM. *P* values shown are adjusted using the Bonferroni correction for multiple comparisons. Only significant differences between groups are shown.

**Table 4:** HC and mild AD performance in the behavioral task of the ERP test

Behavioral measure	HC	AD
Button press accuracy (%)	94.1 ± 1.1	82.2 ± 2.3^∗∗^
False alarms (%)	1.1 ± 0.2	4.9 ± 1.1^∗∗^
Median reaction time (ms)	458.6 ± 11.4	499.5 ± 12.6^∗^

Abbreviations: HC, healthy controls; AD, mild Alzheimer's disease; ERP, event-related potentials; SEM, standard error of the mean.

NOTE. Data are represented as mean ± SEM. *P < .05 and **P < .01 compared with HC after Bonferroni correction.

## Supplementary data

Supplemental Fig. 1 Differences in ERP amplitude for standard, target, and distractor stimuli in mild AD versus HC subjects. Topographies are represented in 100-ms averages going forward. Abbreviations: ERP, event-related potentials; AD, Alzheimer's disease; HC, healthy control.
