# Supplementary figures and images for: A clinical trial to validate event-related potential markers of Alzheimer's disease in outpatient settings
Source: Alzheimers Dement (Amst). 2015 Oct 2;1(4):387–94. doi: 10.1016/j.dadm.2015.08.004 (PMC4879492; doi:10.1016/j.dadm.2015.08.004)

## Slide 1
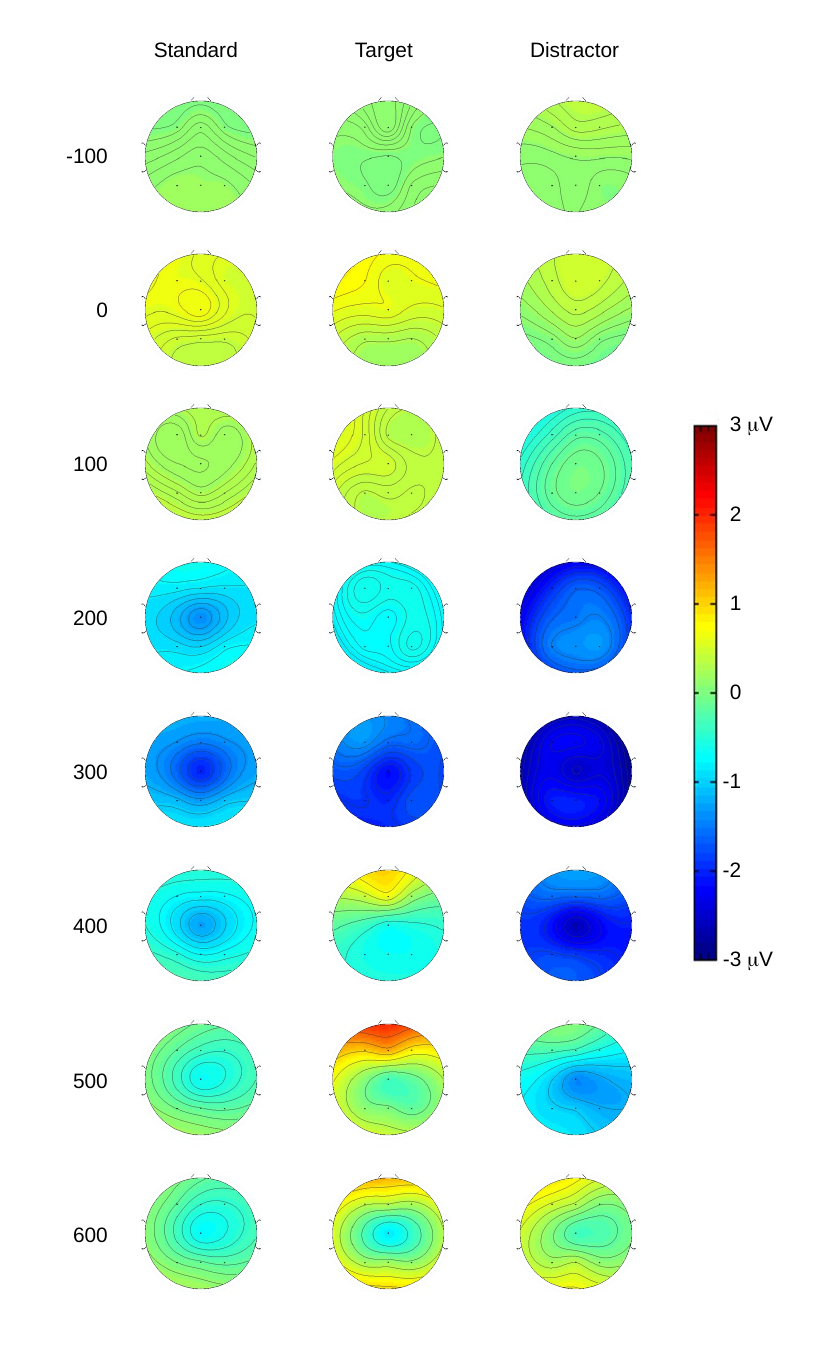

Standard
Target
Distractor
-100
0
3 mV
2
1
0
-1
-2
-3 mV
100
200
300
400
500
600

Supplement: Supplemental Fig. 1 — Differences in ERP amplitude for standard, target, and distractor stimuli in mild AD versus HC subjects. Topographies are represented in 100-ms averages going forward. Abbreviations: ERP, event-related potentials; AD, Alzheimer's disease; HC, healthy control. [file mmc1.pptx]
