# Supplementary material for: A clinical trial to validate event-related potential markers of Alzheimer's disease in outpatient settings
Source: Alzheimers Dement (Amst). 2015 Oct 2;1(4):387–94. doi: 10.1016/j.dadm.2015.08.004 (PMC4879492; doi:10.1016/j.dadm.2015.08.004)
Supplement: Supplemental Table 1 [file mmc2.docx]

| **Supplemental Table 1**  Correlation Matrix of ERP features. | | | | | | | | | | | | | | | | | | | |
| --- | --- | --- | --- | --- | --- | --- | --- | --- | --- | --- | --- | --- | --- | --- | --- | --- | --- | --- | --- |
|  |  | 1 | 2 | 3 | 4 | 5 | 6 | 7 | 8 | 9 | 10 | 11 | 12 | 13 | 14 | 15 | 16 | 17 | 18 |
| 1 | P50 Std Av. Ampl. | 1 |  |  |  |  |  |  |  |  |  |  |  |  |  |  |  |  |  |
| 2 | N100 Std Ampl. | 0.09 | 1 |  |  |  |  |  |  |  |  |  |  |  |  |  |  |  |  |
| 3 | N100 Std Av. Ampl. | 0.11 | **0.95** | 1 |  |  |  |  |  |  |  |  |  |  |  |  |  |  |  |
| 4 | P200 Std Ampl. | 0.12 | -0.31 | -0.20 | 1 |  |  |  |  |  |  |  |  |  |  |  |  |  |  |
| 5 | N100 Tgt Ampl. | 0.01 | **0.83** | **0.81** | -0.31 | 1 |  |  |  |  |  |  |  |  |  |  |  |  |  |
| 6 | N100 Tgt Av. Ampl. | 0.00 | **0.80** | **0.82** | -0.22 | **0.95** | 1 |  |  |  |  |  |  |  |  |  |  |  |  |
| 7 | N200 Tgt Ampl. | 0.06 | 0.19 | 0.25 | 0.19 | 0.24 | 0.29 | 1 |  |  |  |  |  |  |  |  |  |  |  |
| 8 | N200 Tgt Lat. | 0.02 | 0.03 | 0.01 | 0.01 | 0.09 | 0.08 | 0.00 | 1 |  |  |  |  |  |  |  |  |  |  |
| 9 | N200 Tgt Av. Ampl. | 0.11 | 0.05 | 0.13 | 0.36 | 0.09 | 0.15 | **0.85** | -0.15 | 1 |  |  |  |  |  |  |  |  |  |
| 10 | P3b Tgt Ampl. | 0.06 | 0.13 | 0.16 | 0.07 | 0.06 | 0.09 | 0.28 | -0.35 | 0.50 | 1 |  |  |  |  |  |  |  |  |
| 11 | P3b Tgt Lat. | 0.02 | 0.11 | 0.08 | -0.13 | 0.16 | 0.15 | -0.22 | 0.34 | -0.27 | -0.35 | 1 |  |  |  |  |  |  |  |
| 12 | Slow Wave Lat. | 0.17 | 0.05 | 0.12 | 0.03 | 0.06 | 0.06 | 0.22 | 0.03 | 0.25 | 0.16 | 0.16 | 1 |  |  |  |  |  |  |
| 13 | P50 Dtr Ampl. | 0.41 | -0.29 | -0.23 | 0.36 | -0.35 | -0.33 | -0.04 | -0.09 | 0.09 | 0.02 | -0.07 | -0.04 | 1 |  |  |  |  |  |
| 14 | P50 Dtr. Lat. | 0.34 | 0.17 | 0.18 | -0.01 | 0.16 | 0.17 | 0.14 | -0.06 | 0.12 | 0.02 | 0.08 | 0.08 | 0.04 | 1 |  |  |  |  |
| 15 | N100 Dtr. Ampl. | 0.15 | **0.64** | **0.62** | -0.14 | **0.63** | **0.60** | 0.25 | 0.12 | 0.11 | 0.00 | 0.21 | 0.10 | -0.11 | 0.24 | 1 |  |  |  |
| 16 | N100 Dtr. Av. Ampl. | 0.23 | **0.63** | **0.64** | -0.09 | **0.61** | **0.61** | 0.26 | 0.10 | 0.13 | -0.02 | 0.16 | 0.06 | -0.01 | 0.29 | **0.93** | 1 |  |  |
| 17 | P3a Dtr. Ampl. | 0.00 | 0.01 | 0.03 | 0.20 | -0.05 | -0.01 | 0.16 | -0.28 | 0.28 | **0.58** | -0.37 | -0.05 | 0.09 | 0.00 | 0.03 | 0.01 | 1 |  |
| 18 | P3a Dtr. Av. Ampl. | -0.07 | 0.01 | 0.02 | 0.18 | -0.07 | -0.07 | 0.18 | -0.21 | 0.27 | **0.56** | -0.34 | -0.07 | 0.09 | -0.08 | 0.05 | 0.02 | **0.92** | 1 |
| Abbreviations: Std, Standard; Tgt, Target; Dtr, Distractor; Ampl., Amplitude; Lat, Latency; Av. Ampl, Average Amplitude.    NOTE: Data shown are Pearson correlation values. Only correlations between features that were significantly different in mild AD vs. healthy controls are shown. Correlation values above 0.50 are in bold. | | | | | | | | | | | | | | | | | | | |
